# Differing associations between Aβ accumulation, hypoperfusion, blood–brain barrier dysfunction and loss of PDGFRB pericyte marker in the precuneus and parietal white matter in Alzheimer's disease

**DOI:** 10.1177/0271678X17690761

**Published:** 2017-02-02

**Authors:** J Scott Miners, Isabel Schulz, Seth Love

**Affiliations:** Dementia Research Group, Institute of Clinical Neurosciences, School of Clinical Sciences, University of Bristol, Bristol, UK

**Keywords:** Alzheimer's disease, blood–brain barrier, cerebral hypoperfusion, fibrinogen, pericyte

## Abstract

Recent studies implicate loss of pericytes in hypoperfusion and blood–brain barrier (BBB) leakage in Alzheimer's disease (AD). In this study, we have measured levels of the pericyte marker, platelet-derived growth factor receptor-β (PDGFRB), and fibrinogen (to assess blood–brain barrier leakage), and analyzed their relationship to indicators of microvessel density (von Willebrand factor level), ante-mortem oxygenation (myelin-associated glycoprotein:proteolipid protein-1 ratio and vascular endothelial growth factor level), Aβ level and plaque load, in precuneus and underlying white matter from 49 AD to 37 control brains. There was reduction in PDGFRB and increased fibrinogen in the precuneus in AD. These changes correlated with reduction in oxygenation and with plaque load. In the underlying white matter, increased fibrinogen correlated with reduced oxygenation, but PDGFRB level was unchanged. The level of platelet-derived growth factor-ββ (PDGF-BB), important for pericyte maintenance, was increased in AD but mainly in the insoluble tissue fraction, correlating with insoluble Aβ level. Loss of the PDGFRB within the precuneus in AD is associated with fibrinogen leakage and reduced oxygenation, and related to fibrillar Aβ accumulation. In contrast, fibrinogen leakage and reduced oxygenation of underlying white matter occur independently of loss of PDGFRB, perhaps secondary to reduced transcortical perfusion.

## Introduction

Histological and ultrastructural examination of human post-mortem brain tissue has shown evidence of pericyte degeneration in AD,^1–4^ and blood–brain barrier (BBB) breakdown, indicated by the extravasation of serum proteins such as fibrinogen, has also been widely reported.^3–7^ Pericyte loss and BBB breakdown were found to be associated with higher Aβ level and to be exacerbated in *APOE* ε4 carriers.^3,4^ In the present study, we have used biochemical methods to investigate the relationships between the pericyte marker PDGFRB,^4,8,9^ fibrinogen, cerebral hypoperfusion, *APOE* genotype, Braak tangle stage, and the pericyte trophic protein PDGF-BB, by analysis of the precuneus and underlying white matter from post-mortem brain tissue in AD and control brains.

Cerebral blood flow declines within the medial parietal cortex (precuneus) early in the development of Alzheimer's disease (AD). This is the first affected region of brain to show hypoperfusion in AD, up to 10 years before clinical symptoms.^10–16^ Reduced cerebral blood flow predicts the onset of dementia in AD^17^ and occurs well before behavioral or pathological alterations in animal models.^18–20^ It causes ischemic damage and may exacerbate AD pathology by increasing the production and reducing the clearance of Aβ (reviewed in Chui et al.^21^). Conversely, there is evidence that the accumulation of Aβ reduces cerebral blood flow not only through the development of cerebral amyloid angiopathy (CAA) but also by inducing both chronic vasoconstriction and interfering with autoregulation and neurovascular coupling (reviewed in Love and Miners^22^ and Miners et al.^23^).

We previously reported that chronic reduction in oxygenation of brain tissue could be quantified by comparison of the levels of two myelin proteins: myelin-associated glycoprotein (MAG), which is highly susceptible to reduced tissue oxygenation, and proteolipid protein-1 (PLP-1), which is relatively resistant.^24–27^ Both proteins are stable under post-mortem conditions and, as they have half-lives of several months, a decline in MAG:PLP1 reflects reduced oxygenation of oligodendrocytes over a sustained period prior to death (reviewed in Love and Miners^22,28^). Reduced oxygenation of the precuneus is evident at an early stage of AD (i.e. Braak tangle stage III-IV disease),^26^ and correlates strongly with the level of endothelin-1 (EDN1), a potent vasoconstrictor, which is increased in AD,^26,27,29^ probably as a consequence of Aβ42-mediated upregulation of endothelin-converting enzyme-2 (ECE-2),^30^ but Aβ40-mediated upregulation of ECE-1 may also contribute.^29^

Several animal models have provided mechanistic insights into the relationship between neurovascular dysfunction and AD. Deletion of the *Meox2* gene in mice resulted in a perfusion deficit that was associated with increased Aβ level (due to impeded LRP-1-mediated clearance).^31^ Mice deficient in platelet-derived growth factor receptor-β (Pdgfrβ^+/−^), a specific marker of pericytes,^32^ showed age-related loss of pericytes, associated with BBB breakdown, impaired neurovascular coupling, reduced capillary density and reduced cerebral blood flow.^9^ Accelerated progression of AD-related pathology and neuronal loss was observed when Pdgfrβ^+/−^ mice were crossed with APP^sw/0^ transgenic mice.^33^ BBB breakdown and impaired interstitial drainage of Aβ were thought to be responsible for the accelerated neurodegeneration, as neuronal damage was more marked in these mice than in those with a perfusion deficit alone.

Imaging studies have provided additional evidence linking pericyte loss, BBB breakdown and cerebral hypoperfusion in the early stages of AD. In 21 patients with no cognitive impairment, 21 with mild cognitive impairment and 19 patients with multiple sclerosis, imaging revealed that BBB leakage within the hippocampus was a feature of normal aging but was exacerbated in the MCI group.^34^ The severity of BBB leakage correlated with the CSF level of soluble PDGFRB, a marker of pericyte injury.^35^ Van de Haar et al.^36,37^ examined a small cohort of patients with early AD and age-matched controls and found that BBB leakage in early AD was associated with cognitive decline and reduced cerebral blood flow. The precise interrelationship between these neurovascular abnormalities in early AD remains unclear. In clinical and experimental studies of acute stroke, hypoperfusion is closely followed by disruption of the BBB.^38,39^ However, as noted above,^9^ pericyte loss can itself lead to both BBB leakage and hypoperfusion.

In the present study, we have analyzed the relationship between pericyte loss, BBB leakage and hypoperfusion in both the precuneus, as it is a region of early and consistent vascular dysfunction in AD, and the underlying white matter, which is perfused by perforating arterioles that pass through the precuneus. Present findings reveal a strong association between Aβ plaque load, loss of pericyte protein PDGFRB, elevated fibrinogen and hypoperfusion in the cortex in AD. However, in the underlying white matter there is fibrinogen accumulation and hypoperfusion in the absence of loss of PDGFRB. Our findings suggest that fibrillar Aβ accumulation plays a key role in pericyte degeneration in human brain tissue and highlight important differences between cerebral cortex and white matter in the pathophysiology of neurovascular dysfunction in early AD.

## Materials and methods

### Case selection

Brain tissue was obtained from the South West Dementia Brain Bank, University of Bristol, UK, with ethical approval from NRES committee South West-Central Bristol, UK (NRES approval 08/H0106/28 + 5). The brains had been dissected within 72 h of death. The right cerebral cortex had been fixed in buffered formalin for three weeks before the tissue was processed and paraffin blocks were taken for pathological assessment. The left cerebral hemisphere had been sliced and frozen at −80 ℃. We studied 49 brains from patients with AD (ages, mean 77.5 y, SD 8.2 y) with post-mortem delays of 4 to 72 h (mean 31.4 h, SD 19.3 h) and 37 control brains (ages 58 to 94 y (mean 79.8 y, SD 8.9 y) with post-mortem delays from 3 to 67 h (mean 32.7 h, SD 16.3 h). All of the brains had been subjected to detailed neuropathological assessment. In those from patients with AD, the diagnosis had been made according to the NIA-AA guidelines.^40^ Control brains were from people with no history of dementia, few or absent neuritic plaques, a Braak tangle stage of III or less and no other neuropathological abnormalities. The demographic data, neuropathological findings, and MRC identifier numbers in this cohort are summarized in Supplementary Tables 1 and 2.

The cohort overlapped that of a previous study of several determinants of perfusion of the precuneus^26^ and parietal white matter^24^ in AD, vascular dementia, and control brains. Measurements of MAG:PLP1,^24,26^ VEGF,^27^ and soluble and insoluble Aβ40 and Aβ42^41^ were previously reported. Aβ plaque load had been measured by determining the area fraction of cerebral cortex immunopositive for Aβ. Small vessel disease (SVD) had been scored on a 4-point semi-quantitative scale as previously described,^25^ according to the extent of thickening of the arteriolar walls and associated narrowing of the vessel lumina: 0 = normal vessel wall thickness, 1 = slightly increased thickness, 2 = moderately increased thickness, and 3 = markedly increased thickness such that for many arterioles the diameter of the lumen was <50% of the outer diameter of the blood vessel. CAA had also been previously graded semi-quantitatively on a 4-point scale by a method adapted from that of Chalmers et al.^42^ and Olichney et al.,^43^ ranging from “0” for vessels devoid of amyloid to “3” for extensive vascular deposition.

### Preparation of brain tissue

Frozen tissue was dissected from the left medial parietal cortex (Brodmann area 7) and separate samples were dissected from the underlying parietal white matter. Biochemical analyses were performed on 200 mg samples of the dissected tissue that were homogenized in a Precellys homogenizer (Stretton Scientific, Derbyshire, UK) and extracted in 1% sodium dodecyl sulfate lysis buffer or guanidine-HCl, as previously described^24,26,27^ and then aliquoted and stored at −80℃ until required. All measurements were made in duplicate and the mean determined.

### Measurement of PDGFRB

PDGFRB level was measured by sandwich ELISA (duoset, Cat no DYC385, R&D systems, Oxford, UK). High-binding capacity clear 96-well plates (Costar EIA plates, R&D systems, Oxford, UK) were coated overnight at room temperature with anti-human PDGFRB capture antibody (diluted to 6 µg/ml in PBS). The plate was washed five-times in PBS:0.05% tween-20 and blocked for 2 h in PBS:1% bovine serum albumin (Sigma Aldrich, Dorset, UK) at room temperature. Following a further wash step, brain tissue samples at (2 μl + 98 μl PBS) or recombinant human PDGFR-β (16,000 − 250 pg/ml) were incubated for 2 h at room temperature without shaking. The plate was washed and biotinylated anti-human PDGFRB detection antibody (diluted to 0.5 ug/ml in PBS) was added for 2 h at room temperature without shaking. The plate was washed and incubated for 20 min at room temperature with streptavidin:HRP (diluted 1 in 200 in PBS) (R&D systems, UK) and then washed and incubated in the dark with 3,3′,5,5′-tetramethylbenzidine (TMB) substrate (R&D systems, UK) for 15 min. Absorbance was read at 450 nM following the addition of 2 N sulfuric acid, in a FLUOstar OPTIMA plate reader (BMG labtech, Aylesbury, UK). The absolute concentration of PDGFRB was interpolated from the standard curve for each case, derived from duplicate measurement of the recombinant PDGFRB.

To confirm the specificity of the assay, we performed immunofluorescent labeling of sections of parietal cortex and white matter with the PDGFRB detection antibody used in the ELISA, in combination with antibody to smooth muscle actin or Von Willebrand factor (vWF) (see supplementary information for methodological details). This confirmed the finding of Craggs et al.^44^ that PDGFRB is largely restricted to cells associated with capillaries (Supplementary Figure 1). There was negligible overlap of PDGFRB immunofluorescent signal with that of smooth muscle actin in the tunica media of adjacent arterioles, and limited overlap of the PDGFRB signal with that of vWF in the underlying endothelium.

**Figure 1.**
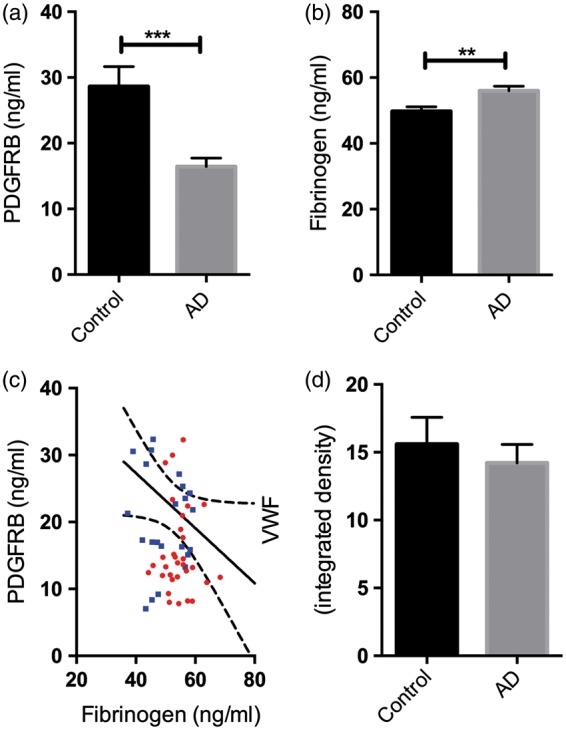
Platelet-derived growth factor receptor-β (PDGFRB) loss and blood–brain barrier (BBB) breakdown in the precuneus in AD. (a) Bar chart showing reduced PDGFRB in AD compared to age-matched controls. (b) Bar chart showing increased fibrinogen level in AD compared to age-matched controls. (c) Scatterplot showing a trend toward a negative correlation between PDGFRB and fibrinogen level in the precuneus (r = −0.25, *P* = 0.054). Each point in the scatterplot indicates a single AD (red circle) or control (blue square) brain. The best-fit linear regression line and 95% confidence interval are superimposed. (d) The level of von Willebrand factor (vWF) in the precuneus did not differ significantly between AD and control brains. The bars indicate the mean and SEM. **P < 0.001, ***P < 0.0001.

### Measurement of fibrinogen

Fibrinogen content was measured in brain tissue homogenates by use of a commercially available sandwich ELISA (Human Fibrinogen ELISA kit, Cat no EH3057, Wuhan Fine Biological Technology Co, Wuhan City, Hubei Province, China). The plate was pre-coated with an anti-human fibrinogen capture antibody. Recombinant human fibrinogen or brain tissue homogenate (50 μl + 50 μl proprietary dilution buffer) was incubated at 37℃ for 90 min. The plate was washed five times in PBS:0.05% tween-20 and biotinylated-detection antibody added at 37℃ for 60 min. Bound antibody was detected as above (see Measurement of PDGFRB). The absolute concentration of fibrinogen was interpolated from measurements of serially diluted recombinant human fibrinogen (600–9.375 ng/ml).

### Measurement of hemoglobin

A colorimetric assay kit (Caymen Chemicals, Cat No 700540) (Ann Arbor, MI, USA) was used according to the manufacturer's instructions to measure the hemoglobin level in the brain tissue homogenates. The proprietary hemoglobin detection reagent was incubated with tissue homogenates and absorbance measured at 560–590 nM in a FLUOstar OPTIMA plate reader. Hemoglobin content was determined by interpolation from measurements of serial dilutions of hemoglobin (0.4–0.016 g/dl) and calculated using the following equation

Hemoglobin(g/dl)=(correctedsampleabsorbance-(y-intercept)/slope))×10×sampledilution.

### Measurement of vWF

vWF level in brain tissue homogenates was determined by dot blot as previously described.^24,27^ Samples were diluted in tris-buffered saline (TBS) (1 in 800) and blotted onto nitrocellulose membrane (GE Healthcare, St. Giles, UK) for 1 h at room temperature. The membrane was blocked in 5% non-fat dried milk protein (NFDMP) in TBS at 4℃ overnight, washed, and then incubated for 1 h with polyclonal rabbit anti-human VWF, (0.3 µg/ml) (Dako, Glostrup, Denmark) at room temperature with agitation. The membrane was washed then incubated with anti-rabbit peroxidase-conjugated secondary antibody (Vector Laboratories, Burlingame, CA, USA) in 5% NFDMP diluted in 0.3% TBS-T for 1 h at room temperature with agitation. The membrane was again washed and then developed using chemiluminescent ECL substrate (Millipore, Billerica, MA, USA) according to the manufacturer's guidelines. Image-J was used to measure the integrated density of each blot. Serial dilutions of a standard reference brain tissue homogenate were used to control for any blot-to-blot variation. We previously demonstrated that vWF is stable under conditions of simulated post-mortem delay for up to 72 h at 4℃ or RT and that vWF level, measured by dot blot, is an excellent indicator of microvessel density.^24,45^

### Measurement of PDGF-BB

PDGFBB level in 1% sodium dodecyl sulfate lysis buffer or guanidine-HCl extracts of the homogenized brain tissue was measured by sandwich ELISA (duoset, Cat no DY220, R&D systems, Oxford, UK). High-binding capacity clear 96-well plates (Costar EIA plates, R&D systems, Oxford, UK) were coated overnight at room temperature with anti-human PDGF-BB capture antibody (diluted to 0.4 µg/ml in PBS). The plate was washed five-times in PBS:0.05% tween-20 and blocked for 2 h in PBS:1% bovine serum albumin (Sigma Aldrich, Dorset, UK) at room temperature. Following a further wash step, brain tissue samples at (5 μl + 95 μl PBS) or recombinant human PDGFRB (2000 – 31.25 pg/ml) were incubated for 2 h at room temperature without shaking. The plate was washed and biotinylated anti-human PDGF-BB detection antibody (diluted to 0.4 μg/ml in PBS) was added for 2 h at room temperature without shaking. The plate was washed and incubated for 20 min at room temperature with streptavidin:HRP (diluted 1:200 in PBS) (R&D systems, UK) and then washed and incubated in the dark with 3,3′,5,5′-TMB substrate (R&D systems, UK) for 15 min. Absorbance was read at 450 nM following the addition of 2 N sulfuric acid, in a FLUOstar OPTIMA plate reader (BMG labtech, Aylesbury, UK). The absolute concentration of PDGF-BB was interpolated from the standard curve for each case, derived from duplicate measurement of the recombinant PDGF-BB.

### Statistical analysis

Unpaired two-tailed t-tests or ANOVA with Bonferroni post-hoc analysis were used for comparisons between groups, and Pearson's or Spearman's test to assess linear or rank order correlation, as appropriate, with the help of SPSS version 16 (SPSS, Chicago) and GraphPad Prism version 6 (GraphPad Software, La Jolla, CA). *P*-values < 0.05 were considered statistically significant. We also used Wizard version 1.8.22 (http://www.wizardmac.com/) to perform multivariable regression analysis of the association of PDGFRB, fibrinogen, and PDGF-BB with age, gender, post-mortem delay, and diagnosis of AD.

## Results

### PDGFRB loss, BBB breakdown and reduced microvessel density in the precuneus in AD

PDGFRB level was significantly reduced in the precuneus in AD compared to age-matched controls (*P* = 0.0002) (Figure 1(a)), and fibrinogen level, which rises with BBB breakdown^3,4,6^ was significantly elevated (*P* = 0.0026) (Figure 1(b)). Fibrinogen level tended to vary inversely with PDGFRB level (r = −0.25, *P* = 0.054) (Figure 1(c)), suggesting that BBB breakdown was related to lower pericyte content within the precuneus. There was a small, non-significant reduction in the level of vWF in the precuneus in AD (Figure 1(d)). We previously showed that vWF level is reduced in mid-frontal cortex in AD but was unchanged or elevated in regions of cerebrum that have less Aβ pathology, such as the thalamus.^27^

**Figure 2.**
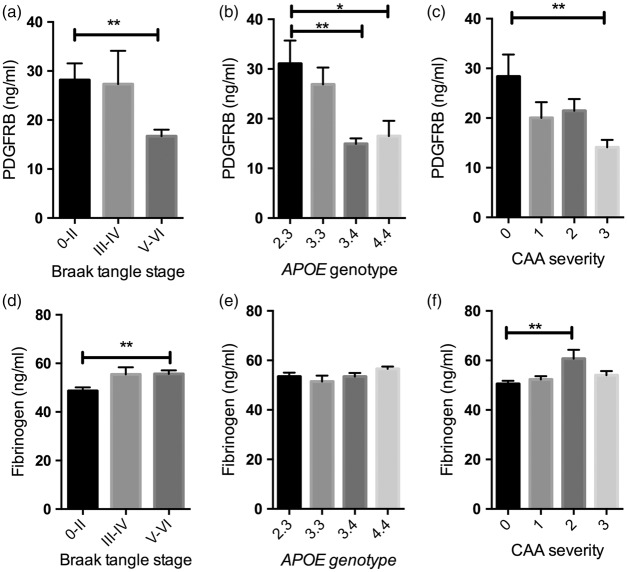
Platelet-derived growth factor receptor-β (PDGFRB) loss and blood–brain barrier (BBB) breakdown in relation to disease severity (i.e. Braak tangle stage), *APOE* genotype, and cerebral amyloid angiopathy (CAA) in the precuneus in AD. Bar charts showing (a) reduced PDGFRB in Braak tangle stage V–VI (end-stage) compared to Braak tangle stage 0–II. (*P* < 0.01) (b) reduced PDGFRB level in *APOE ε*3.4 (*P* < 0.01) and *APOE ε*4.4 (*P* < 0.05) compared with *APOE ε*2.3 individuals and (c) reduced PDGFRB level in severe CAA compared to absent CAA (*P* < 0.01). Bar charts showing (d) increased fibrinogen level in Braak tangle stage V–VI (end stage) compared to Braak stage 0–II (*P* < 0.01), (e) no significant difference in fibrinogen level in relation to *APOE* genotype and (f) increased fibrinogen level in moderate CAA compared to absent CAA (*P* < 0.01). The bars indicate the mean and SEM. CAA severity scores adapted from Olichney et al.:^42,43^ 0 = absent, 1 = mild, 2 = moderate, 3 = severe.

When grouped according to Braak tangle stage, PDGFRB level was significantly lower (Figure 2(a)) and fibrinogen level higher (Figure 2(d)) in Braak tangle stage V–VI (end-stage) disease than in Braak stage 0–II brains (*P* < 0.01 for both). The differences in PDGFRB level and fibrinogen between Braak stage 0–II and III–IV, and between Braak stage III–IV and V–VI were not statistically significant. PDGFRB level was significantly lower in *APOE ε*3.4 (*P* < 0.01) and *APOE ε*4.4 (*P* < 0.05) brains than in those with an *APOE ε*2:3 genotype (Figure 2(b)) but did not vary between *APOE ε*2.3 and *ε*3.3. PDGFRB level was significantly lower in severe CAA than in brains without CAA (*P* < 0.01) (Figure 2(c)). Fibrinogen level did not vary significantly with *APOE* genotype (Figure 2(e)) but was increased in moderate CAA (*P* < 0.01) (Figure 2(f)).

Finally, to ensure that alterations in fibrinogen level within the brain were not caused by differences in blood content, we compared fibrinogen level after adjusting the values (i) for hemoglobin content, and (ii) for microvessel content/vWF level. The hemoglobin-adjusted values remained higher in AD but the difference did not quite reach significance (*P* = 0.09) (Supplementary Figure 2(a)). The vWF-adjusted fibrinogen values were also elevated in AD but again did not reach statistical significance (Supplementary Figure 2(b)).

There was no significant association between PDGFRB in the precuneus, and gender, age or post-mortem delay in either the AD or control cohort, or when all cases were combined. Fibrinogen in the precuneus did not vary with gender or age but did increase slightly with post-mortem delay in the AD (*P* = 0.018, coefficient of correlation 0.163) and combined cohorts (*P* = 0.010, coefficient of correlation 0.148). However, the association of PDGFRB with AD remained significant (*P* = 0.004) after incorporating post-mortem delay into a multivariable regression model (results not shown).

### PDGFRB loss and fibrinogen accumulation in AD, associated with cerebral hypoperfusion of the precuneus

**Figure 3.**
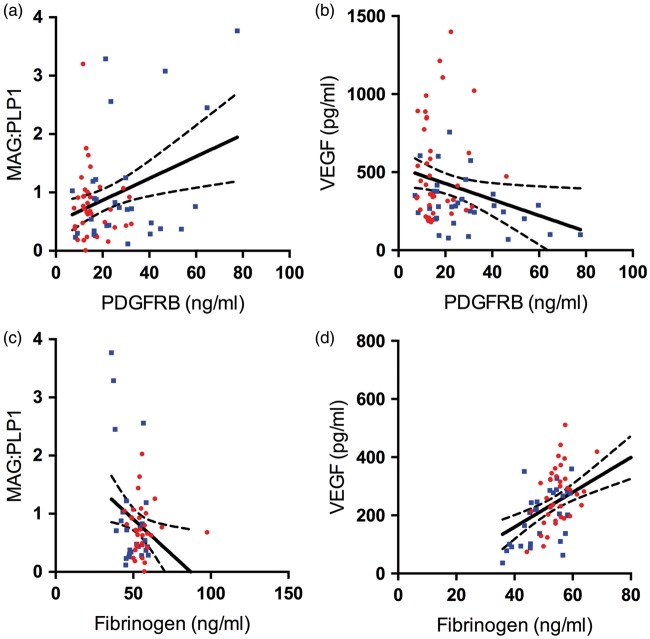
Platelet-derived growth factor receptor-β (PDGFRB) loss and blood–brain barrier breakdown are associated with hypoperfusion of the precuneus. (a) Scatterplot showing a strong positive correlation between PDGFRB and MAG:PLP1 (r = 0.24, *P* = 0.006), i.e. the lowest PDGFRB levels were in samples with least preservation of MAG relative to PLP1. (b) Scatterplot showing a negative correlation between PDGFRB level and VEGF level in the precuneus (r = −0.26, *P* = 0.029), i.e. the lowest PDGFRB levels were in samples with greatest elevation in VEGF. (c) Scatterplot showing a negative correlation between fibrinogen level and MAG:PLP1 (r = −0.30, *P* = 0.022) and (d) Scatterplot showing a strongly positive correlation between fibrinogen and VEGF level (r = 0.49, *P* < 0.0001). Each point in the scatterplots indicates a single AD (red circle) or control (blue square) brain. The best-fit linear regression lines and 95% confidence intervals are superimposed.

We previously reported that MAG:PLP1 was reduced in the precuneus from an early stage of AD, indicating a perfusion deficit with respect to the energy requirements.^26^ In the present study, PDGFRB level correlated positively with MAG:PLP1 (r = +0.34, *P* = 0.006) and negatively with VEGF level (r = −0.26, P = 0.029), providing evidence that pericyte loss is associated with cerebral hypoperfusion (Figure 3(a) and (b)). Fibrinogen level correlated negatively with MAG:PLP (r = −0.30, *P* = 0.022) (Figure 3(c)) and positively with VEGF (r = 0.49, *P* < 0.0001) (Figure 3(d)) indicating that BBB breakdown is also associated with chronic cerebral hypoperfusion.

### PDGFRB loss and BBB breakdown, associated with accumulation of parenchymal Aβ within the precuneus

**Figure 4.**
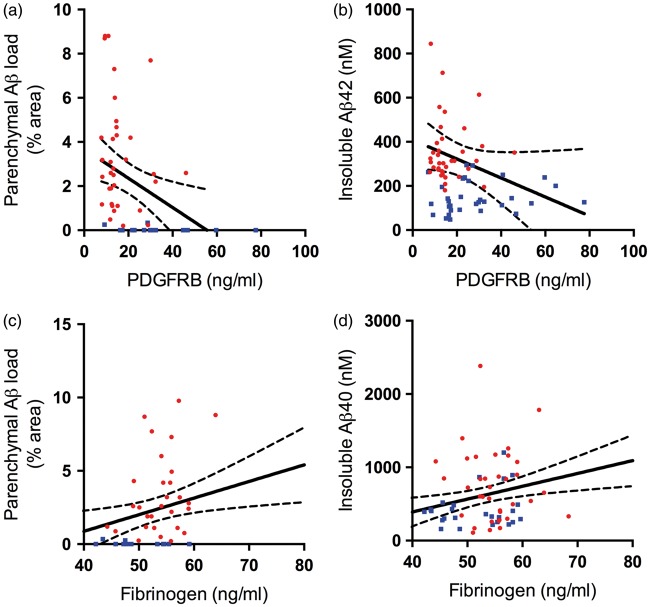
Platelet-derived growth factor receptor-β (PDGFRB) loss and BBB breakdown in the precuneus in AD are related to Aβ level. (a–b) Scatterplots showing negative correlation between PDGFRB and Aβ plaque load (area fraction of cortex immunopositive for Aβ) (r = −0.36, *P* = 0.022) and insoluble Aβ42 level (r = −0.26, *P* = 0.029). (c–d) Scatterplots showing positive correlation between fibrinogen level and Aβ plaque load (r = 0.37, *P* = 0.015) and insoluble Aβ40 (r = 0.35, *P* = 0.006). Each point in the scatterplots indicates a single AD (red circle) or control (blue square) brain. The best-fit linear regression lines and 95% confidence intervals are superimposed.

PDGFRB level correlated negatively with parenchymal Aβ plaque load (r = −0.36, *P* = 0.010) (Figure 4(a)). PDGFRB level also declined with increasing insoluble Aβ42 but this relationship did not reach statistical significance (r = −0.20, *P* = 0.097) (Figure 4(b)). Fibrinogen level correlated positively with parenchymal Aβ load (r = 0.37, *P* = 0.015) (Figure 4(c)) and insoluble Aβ40 (r = 0.33, *P* = 0.006) (Figure 4(d)) but not Aβ42. No correlations were observed between either PDGFRB or fibrinogen with soluble species of Aβ40 or Aβ42.

### Hypoperfusion of cerebral white matter in AD, associated with fibrinogen accumulation without reduction in PDGFRB

**Figure 5.**
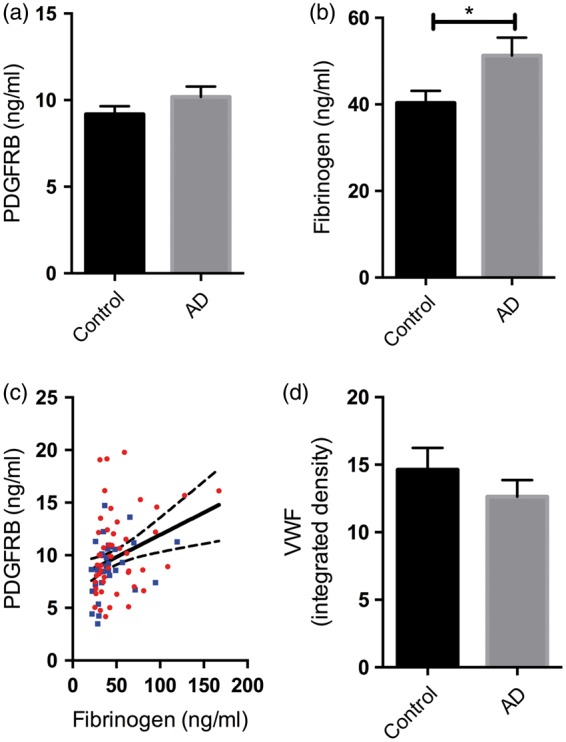
Blood–brain barrier (BBB) breakdown in parietal white matter in AD not associated with pericyte loss. (a) There was no significant change in platelet-derived growth factor receptor-β (PDGFRB) in the parietal white matter in AD. (b) Bar chart showing increased fibrinogen level in white matter in AD compared to age-matched controls. (c) Scatterplot showing highly significant positive correlation between PDGFRB and fibrinogen level in white matter (r = 0.36, *P* < 0.0001). Each point indicates a single AD (red circle) or control (blue square) brain. The best-fit linear regression line and 95% confidence interval are superimposed. (d) The level of von Willebrand factor (vWF), a marker of vessel density, was not significantly altered in the parietal white matter in AD. The bars indicate the mean and SEM. *P < 0.05.

In contrast to the precuneus, the white matter did not show significant alteration in PDGFRB in AD (Figure 5(a)). Fibrinogen level in the white matter was significantly higher in AD than controls (*P* = 0.05) (Figure 5(b)) and remained higher after adjustment for hemoglobin but not vWF level (Supplementary Figure 2(c) and (d)). Unlike in the precuneus, in underlying white matter, PDGFRB correlated positively (rather than negatively) with fibrinogen level (r = 0.36, *P* < 0.0001) (Figure 5(c)). Differences between AD and control brains in vWF level in the parietal white matter were not significant (Figure 5(d)). White matter PDGFRB and fibrinogen level did not vary significantly with Braak tangle stage or *APOE* genotype (Supplementary Figure 3).

**Figure 6.**
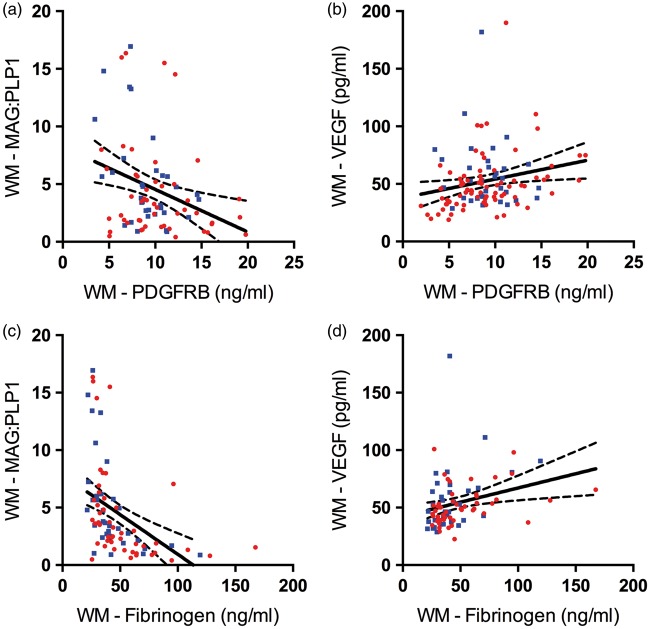
Reduced oxygenation in the parietal white matter in AD associated with blood–brain barrier (BBB) breakdown despite concomitant increase in pericytes. (a) Scatterplot showing strong negative correlation between platelet-derived growth factor receptor-β (PDGFRB) and myelin-associated glycoprotein:proteolipid protein-1 (MAG:PLP1) ratio in white matter (WM) (r = −0.31, *P* = 0.004). (b) Scatterplot showing positive correlation between platelet-derived growth factor receptor-β (PDGFRB) level and vascular endothelial growth factor (VEGF) level (r = 0.19, *P* = 0.019). (c) Scatterplot showing very strong negative correlation between fibrinogen and MAG:PLP1 (r = −0.48, *P* < 0.0001). (d) Scatterplot showing very strong positive correlation between fibrinogen and VEGF level in the white matter (r = 0.42, *P* < 0.0001). Each point in the scatterplots indicates a single AD (red circle) or control (blue square) brain. The best-fit linear regression lines and 95% confidence intervals are superimposed.

We previously reported on chronic hypoperfusion (reduction in MAG:PLP1) in the parietal white matter in AD.^24^ Unlike in the precuneus, PDGFRB level in the white matter correlated negatively with MAG:PLP1 (r = −0.31, *P* = 0.004) (Figure 6(a)) and positively with VEGF (r = 0.19, *P* = 0.019) (Figure 6(b)) indicating an increase in PDGFRB with declining white matter perfusion. Fibrinogen level, however, showed a strong negative correlation with MAG:PLP1 (r = −0.48, *P* < 0.0001) and a strong positive correlation with VEGF (r = 0.42, *P* < 0.0001), suggesting a close relationship between breakdown of the BBB and chronic hypoperfusion in the white matter (Figure 6(c) and (d)).

There was no significant association between PDGFRB and fibrinogen in the white matter, and gender, age or post-mortem delay in either the AD or control cohort, or when all cases were combined.

### Increased PDGF-BB in AD, associated with hypoperfusion, BBB leakiness, and Aβ accumulation

**Figure 7.**
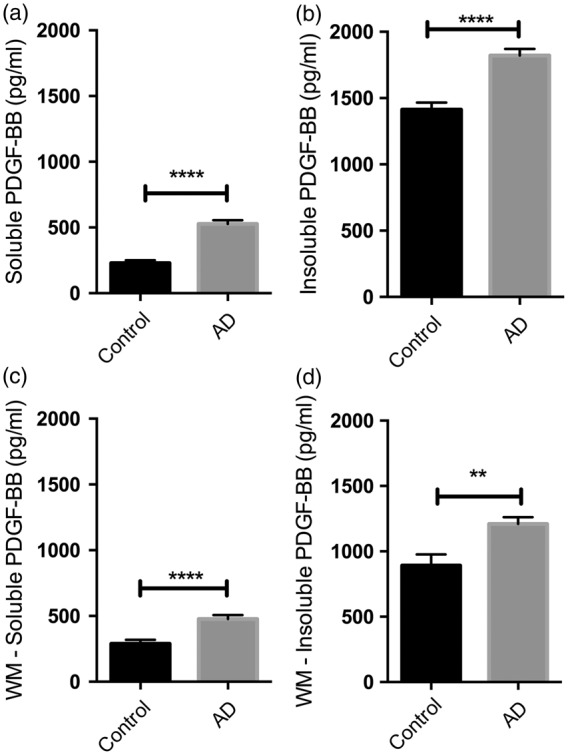
Platelet-derived growth factor-ββ (PDGF-BB) level is increased in the precuneus and underlying white matter in AD, and present in greater quantities in the insoluble fraction. (a–b) Bar charts showing increased PDGF-BB in the precuneus in AD in (a) SDS-extracted soluble (*P* < 0.0001) and (b) guanidine-HCl-extracted insoluble fractions of brain tissue homogenates (*P* < 0.0001). (c–d) Bar charts showing increased PDGF-BB in underlying white in AD in (c) soluble (*P* < 0.0001) and (d) insoluble (*P* = 0.001) fractions. The bars indicate the mean and SEM.

To determine whether the PDGFRB loss in the precuneus was secondary to a decline in PDGF-BB (a key trophic factor for pericytes), we measured the concentration of PDGF-BB in the precuneus and underlying white matter, in both SDS-extracted (soluble) and guanidine-HCl (insoluble) fractions. In AD, the level of PDGF-BB was significantly increased in both regions and in both fractions (*P* < 0.0001 for all comparisons between AD and control brains except in the insoluble white matter fraction where *P* = 0.001) (Figure 7). Of possible relevance, the concentration of PDGF-BB was approximately 3- to 4-fold higher in the insoluble than the corresponding soluble fractions. In the precuneus, PDGF-BB concentration showed highly significant positive correlations with the levels of insoluble Aβ40 (r = 0.43, *P* < 0.01) and Aβ42 (r = 0.40, *P* < 0.01) (Supplementary Table 3). In contrast, PDGF-BB showed weaker negative correlations with Aβ40 (r = −0.298, *P* < 0.05) and Aβ42 (r = −0.212, *P* < 0.05) within the GuHCl-extract in the underlying white matter, which contains much less insoluble Aβ.^41^

There was no significant association between PDGF-BB in the precuneus or white matter, and gender, age or post-mortem delay in either the AD or control cohort, or when all cases were combined.

## Discussion

We previously reported that oxygenation of both the precuneus and the underlying white matter was reduced in the early stages of AD.^26^ Present findings show the reduced oxygenation of the precuneus to be associated with loss of the pericyte protein PDGFRB, and the accumulation of fibrinogen and Aβ (particularly plaque-associated fibrillar Aβ). In contrast, hypoperfusion of the underlying white matter (where the amount of Aβ is orders of magnitude lower than in the precuneus in AD^41^) was associated with fibrinogen accumulation in the absence of any reduction in PDGFRB. The pathophysiological processes that underlie pericyte degeneration, BBB breakdown, and cerebral hypoperfusion are thus region-specific and likely to be influenced by Aβ level. In neither cortex nor white matter could loss of PDGFRB be attributed to a reduced level of PDGF-BB, which was elevated in AD. However, as discussed below, the differential distribution of fibrillar Aβ may have influenced the availability of this trophic factor.

Our findings are consistent with previous reports of an association between pericyte loss and BBB breakdown in AD^34^ and provide further evidence that pericyte loss is related to Aβ level and influenced by *APOE* genotype.^3,4^ At least within the cerebral cortex, there is also a close relationship between PDGFRB content, fibrinogen, and blood flow, in keeping with in vivo evidence that loss of BBB integrity is related to pericyte degeneration, cerebral hypoperfusion,^37^ and cognitive impairment.^36^ Uncertainty persists as to the precise timing of the various vascular abnormalities in AD and the details of the complex causal interrelationships. In Pdgfrβ^+/−^ mice, age-related pericyte loss led to loss of cerebral microvessels, breakdown of the BBB, reduced cerebral blood flow, and neurovascular uncoupling.^9^ Evidence that these vascular abnormalities have the potential to exacerbate AD pathology comes from a study in which APP^sw/0^ mice developed more severe Aβ and tau pathology, neuronal loss, and memory impairment when they were crossed with Pdgfrb^+/−^ mice.^33^

In contrast, several lines of evidence suggest that Aβ contributes to pericyte injury,^33,35,46^ BBB dysfunction,^3,4^ and hypoperfusion.^26,27,47^ In recent years imaging studies, particularly in individuals with familial AD, have shown that Aβ begins to accumulate as early as 20 years before the development of dementia, whereas cerebral hypoperfusion occurs later, 5 to 10 years before disease onset.^11,12^ In vitro studies indicate that Aβ peptides cause pericyte injury and death.^33,35,46^ It is also possible that the damaging effects of Aβ on pericyte survival are partly indirect, through the sequestration of PDGF-BB, upregulation of VEGF, and even through competition for binding to PDGFRB.

PDGF-BB was shown previously to be mainly associated with Aβ plaques in AD brains.^48^ Although we found PDGF-BB to be increased in AD, possibly as a physiological response to reduced tissue oxygenation, most of it was in the insoluble fraction of the tissue, which also contained high levels of Aβ. Indeed, there was a close correlation between the levels of PDGF-BB and Aβ within the insoluble fraction, as might be expected if the PDGF-BB was sequestered by fibrillar Aβ and thus biologically inactive – a mechanism proposed to apply to another vascular trophic factor in AD, VEGF.^49^ VEGF level is significantly increased in AD, as noted in the present study and also previously.^27^ Although VEGF induces vasculogenesis as well as elongation and migration of pericytes, and may promote their interaction with capillaries during development,^50^ VEGF also acts as a negative regulator of pericyte function and maturation of vessels, interferes with the interaction between pericytes and endothelial cells and may promote vascular leakage.^51–53^ A final possibility to consider is whether Aβ may interfere with the binding of PDGF-BB to its pericyte receptor, PDGFRB. Again, the VEGF signaling pathway provides an example of this mechanism, in that Aβ was shown to interfere with the binding of VEGF to VEGF receptor-2.^54^

Data from previous studies suggest that there is loss of pericytes and leakage of fibrinogen at a relatively early stage of AD.^34,36,37^ We found PDGFRB and fibrinogen levels to vary significantly with Braak tangle stage. On pairwise post hoc testing, only differences between Braak stage 0–II and V–VI disease were significantly different. However, our cohort included relatively few cases in the Braak stage III–IV group and there was greater variation between cases in this group than in the 0–II and V–VI groups. Further analysis of larger cohorts is needed to clarify the precise timing of pericyte loss and BBB leakage in relation to tangle pathology and other pathological features of AD.

We found that PDGFRB level was preserved or even increased in the white matter in AD, possibly because there was insufficient fibrillar Aβ to damage pericytes or to sequester PDGF-BB. Factors other than pericyte loss must account for the white matter hypoperfusion and BBB leakage. Most of the perfusion of the white matter derives from transcortical perforating arterioles, and we reported previously that oxygenation of the cerebral white matter declined with increasing levels of the vasoconstrictor EDN1 in the overlying cortex, suggesting that hypoperfusion of the white matter in AD results partly from vasoconstriction of perforating arterioles as they traverse the cortex.^26,47,55^ It is possible that BBB leakage in the white matter is secondary to hypoperfusion.^38,39^ Alternatively, it could reflect upregulation of the plasma kallikrein-kinin system in AD,^56^ leading to increased production of bradykinin, which increases the permeability of the BBB.^57–59^ This merits further study.

Together, our data suggest that pericytes degenerate in regions of brain with elevated fibrillar Aβ, causing or exacerbating breakdown of the BBB, and possibly contributing to loss of microvessels, hypoperfusion, and impaired vascular clearance of Aβ. Factors other than pericyte degeneration are likely to be responsible for hypoperfusion and breakdown of the BBB in the white matter in AD. Further study is needed of the timing, regional distribution, and mechanisms of pericyte degeneration in AD.

## Supplementary Material

Supplementary material

## References

[1] Baloyannis SJ Baloyannis IS The vascular factor in Alzheimer's disease: A study in Golgi technique and electron microscopy. J Neurol Sci 2012; 322: 117–121. 22857991 10.1016/j.jns.2012.07.010

[2] Farkas E Luiten PG Cerebral microvascular pathology in aging and Alzheimer's disease. Prog Neurobiol 2001; 64: 575–611. 11311463 10.1016/s0301-0082(00)00068-x

[3] Halliday MR Rege SV Ma Q et al. Accelerated pericyte degeneration and blood-brain barrier breakdown in apolipoprotein E4 carriers with Alzheimer's disease. J Cereb Blood Flow Metab 2016; 36: 216–227. 25757756 10.1038/jcbfm.2015.44 PMC4758554

[4] Sengillo JD Winkler EA Walker CT et al. Deficiency in mural vascular cells coincides with blood-brain barrier disruption in Alzheimer's disease. Brain Pathol 2013; 23: 303–310. 23126372 10.1111/bpa.12004 PMC3628957

[5] Cortes-Canteli M Mattei L Richards AT et al. Fibrin deposited in the Alzheimer's disease brain promotes neuronal degeneration. Neurobiol Aging 2015; 36: 608–617. 25475538 10.1016/j.neurobiolaging.2014.10.030 PMC4315732

[6] Hultman K Strickland S Norris EH The APOE ∈4/∈4 genotype potentiates vascular fibrin(ogen) deposition in amyloid-laden vessels in the brains of Alzheimer's disease patients. J Cereb Blood Flow Metab 2013; 33: 1251–1258. 23652625 10.1038/jcbfm.2013.76 PMC3734776

[7] Zipser BD Johanson CE Gonzalez L et al. Microvascular injury and blood-brain barrier leakage in Alzheimer's disease. Neurobiol Aging 2007; 28: 977–986. 16782234 10.1016/j.neurobiolaging.2006.05.016

[8] Craggs LJL Fenwick R Oakley AE et al. Immunolocalization of platelet-derived growth factor receptor-β (PDGFR-β) and pericytes in cerebral autosomal dominant arteriopathy with subcortical infarcts and leukoencephalopathy (CADASIL). Neuropath Appl Neuro 2015; 41: 557–570. 10.1111/nan.12188 PMC5098250 25303037

[9] Bell RD Winkler EA Sagare AP et al. Pericytes control key neurovascular functions and neuronal phenotype in the adult brain and during brain aging. Neuron 2010; 68: 409–427. 21040844 10.1016/j.neuron.2010.09.043 PMC3056408

[10] Asllani I Habeck C Scarmeas N et al. Multivariate and univariate analysis of continuous arterial spin labeling perfusion MRI in Alzheimer's disease. J Cereb Blood Flow Metab 2008; 28: 725–736. 17960142 10.1038/sj.jcbfm.9600570 PMC2711077

[11] Benzinger TLS Blazey T Jack CR et al. Regional variability of imaging biomarkers in autosomal dominant Alzheimer's disease. Proc Natl Acad Sci U S A 2013; 110: E4502–E4509. 24194552 10.1073/pnas.1317918110 PMC3839740

[12] Binnewijzend MAA Kuijer JPA Benedictus MR et al. Cerebral blood flow measured with 3D pseudocontinuous arterial spin-labeling MR imaging in Alzheimer disease and mild cognitive impairment: A marker for disease severity. Radiology 2013; 267: 221–230. 23238159 10.1148/radiol.12120928

[13] Dai WY Lopez OL Carmichael OT et al. Mild cognitive impairment and Alzheimer disease: Patterns of altered cerebral blood flow at MR imaging. Radiology 2009; 250: 856–866. 19164119 10.1148/radiol.2503080751 PMC2680168

[14] Langbaum JBS Chen KW Caselli RJ et al. Hypometabolism in Alzheimer-affected brain regions in cognitively healthy Latino individuals carrying the apolipoprotein E ∈4 allele. Arch Neurol 2010; 67: 462–468. 20385913 10.1001/archneurol.2010.30 PMC2943432

[15] Matsuda H Cerebral blood flow and metabolic abnormalities in Alzheimer's disease. Ann Nucl Med 2001; 15: 85–92. 11448080 10.1007/BF02988596

[16] Sakamoto S Ishii K Sasaki M et al. Differences in cerebral metabolic impairment between early and late onset types of Alzheimer's disease. J Neurol Sci 2002; 200: 27–32. 12127672 10.1016/s0022-510x(02)00114-4

[17] Ruitenberg A den Heijer T Bakker SLM et al. Cerebral hypoperfusion and clinical onset of dementia: The Rotterdam study. Ann Neurol 2005; 57: 789–794. 15929050 10.1002/ana.20493

[18] Niwa K Kazama K Younkin L et al. Cerebrovascular autoregulation is profoundly impaired in mice overexpressing amyloid precursor protein. Am J Physiol Heart Circ Physiol 2002; 283: H315–H323. 12063304 10.1152/ajpheart.00022.2002

[19] Niwa K Kazama K Younkin SG et al. Alterations in cerebral blood flow and glucose utilization in mice overexpressing the amyloid precursor protein. Neurobiol Dis 2002; 9: 61–68. 11848685 10.1006/nbdi.2001.0460

[20] Niwa K Porter VA Kazama K et al. Aβ-peptides enhance vasoconstriction in cerebral circulation. Am J Physiol Heart Circ Physiol 2001; 281: H2417–2424. 11709407 10.1152/ajpheart.2001.281.6.H2417

[21] Chui HC Zheng L Reed BR et al. Vascular risk factors and Alzheimer's disease: Are these risk factors for plaques and tangles or for concomitant vascular pathology that increases the likelihood of dementia? An evidence-based review. Alzheimers Research & Therapy 2012; 4: 1. 10.1186/alzrt98 PMC3471388 22182734

[22] Love S Miners JS Cerebrovascular disease in ageing and Alzheimer's disease. Acta Neuropathol 2016; 131: 645–658. 26711459 10.1007/s00401-015-1522-0 PMC4835514

[23] Miners JS Palmer JC Tayler H et al. Abeta degradation or cerebral perfusion? Divergent effects of multifunctional enzymes. Front Aging Neurosci 2014; 6: 238. 25309424 10.3389/fnagi.2014.00238 PMC4160973

[24] Barker R Ashby EL Wellington D et al. Pathophysiology of white matter perfusion in Alzheimer's disease and vascular dementia. Brain 2014; 137: 1524–1532. 24618270 10.1093/brain/awu040 PMC3999715

[25] Barker R Wellington D Esiri MM et al. Assessing white matter ischemic damage in dementia patients by measurement of myelin proteins. J Cereb Blood Flow Metab 2013; 33: 1050–1057. 23532085 10.1038/jcbfm.2013.46 PMC3705431

[26] Miners JS Palmer JC Love S Pathophysiology of hypoperfusion of the precuneus in early Alzheimer's disease. Brain Pathol 2016; 26: 533–541. 26452729 10.1111/bpa.12331 PMC4982069

[27] Thomas TL Miners SJ Kehoe PG et al. Correlation between levels of VEGF and insoluble Aβ(1-42) in the cerebral cortex in Alzheimer's disease. Neuropathol Appl Neurobiol 2015; 41: 22.

[28] Love S Miners JS White matter hypoperfusion and damage in dementia: Post-mortem assessment. Brain Pathol 2015; 25: 99–107. 25521180 10.1111/bpa.12223 PMC8029367

[29] Palmer JC Barker R Kehoe PG et al. Endothelin-1 is elevated in Alzheimer's disease and upregulated by amyloid-β. J Alzheimers Dis 2012; 29: 853–861. 22330820 10.3233/JAD-2012-111760

[30] Palmer JC Baig S Kehoe PG et al. Endothelin-converting enzyme-2 is increased in Alzheimer's disease and up-regulated by Aβ. Am J Pathol 2009; 175: 262–270. 19541930 10.2353/ajpath.2009.081054 PMC2708812

[31] Wu Z Guo H Chow N et al. Role of the MEOX2 homeobox gene in neurovascular dysfunction in Alzheimer disease. Nat Med 2005; 11: 959–965. 16116430 10.1038/nm1287

[32] Winkler EA Bell RD Zlokovic BV Pericyte-specific expression of PDGF β receptor in mouse models with normal and deficient PDGF beta receptor signaling. Mol Neurodegener 2010; 5: 32. 20738866 10.1186/1750-1326-5-32 PMC2936891

[33] Sagare AP Bell RD Zhao Z et al. Pericyte loss influences Alzheimer-like neurodegeneration in mice. Nat Commun 2013, pp. 4. 10.1038/ncomms3932 PMC3945879 24336108

[34] Montagne A Barnes SR Sweeney MD et al. Blood-brain barrier breakdown in the aging human hippocampus. Neuron 2015; 85: 296–302. 25611508 10.1016/j.neuron.2014.12.032 PMC4350773

[35] Sagare AP Sweeney MD Makshanoff J et al. Shedding of soluble platelet-derived growth factor receptor-β from human brain pericytes. Neurosci Lett 2015; 607: 97–101. 26407747 10.1016/j.neulet.2015.09.025 PMC4631673

[36] van de Haar HJ Burgmans S Jansen JF et al. Blood-brain barrier leakage in patients with early Alzheimer disease. Radiology 2016; 281: 527–535. 27243267 10.1148/radiol.2016152244

[37] van de Haar HJ Jansen JF van Osch MJ et al. Neurovascular unit impairment in early Alzheimer's disease measured with magnetic resonance imaging. Neurobiol Aging 2016; 45: 190–196. 27459939 10.1016/j.neurobiolaging.2016.06.006

[38] Garrigue P Giacomino L Bucci C et al. Single photon emission computed tomography imaging of cerebral blood flow, blood-brain barrier disruption, and apoptosis time course after focal cerebral ischemia in rats. Int J Stroke 2016; 11: 117–126. 26763027 10.1177/1747493015607516

[39] Lorberboym M Lampl Y Sadeh M Correlation of 99mTc-DTPA SPECT of the blood-brain barrier with neurologic outcome after acute stroke. J Nucl Med 2003; 44: 1898–1904. 14660714

[40] Montine TJ Phelps CH Beach TG et al. National Institute on Aging-Alzheimer's Association guidelines for the neuropathologic assessment of Alzheimer's disease: a practical approach. Acta Neuropathol 2012; 123: 1–11. 22101365 10.1007/s00401-011-0910-3 PMC3268003

[41] Miners JS Clarke P Love S Clusterin levels are increased in Alzheimer's disease and influence the regional distribution of Aβ. Brain Pathol. 2016. Epub ahead of print 8 July 2016. DOI: 10.1111/bpa.12392. 10.1111/bpa.12392 PMC8029153 27248362

[42] Chalmers K Wilcock GK Love S APOE ∈4 influences the pathological phenotype of Alzheimer's disease by favouring cerebrovascular over parenchymal accumulation of Aβ protein. Neuropathol Appl Neurobiol 2003; 29: 231–238. 12787320 10.1046/j.1365-2990.2003.00457.x

[43] Olichney JM Hansen LA Galasko D et al. The apolipoprotein E ∈4 allele is associated with increased neuritic plaques and cerebral amyloid angiopathy in Alzheimer's disease and Lewy body variant. Neurology 1996; 47: 190–196. 8710076 10.1212/wnl.47.1.190

[44] Craggs LJ Fenwick R Oakley AE et al. Immunolocalization of platelet-derived growth factor receptor-β (PDGFR-β) and pericytes in cerebral autosomal dominant arteriopathy with subcortical infarcts and leukoencephalopathy (CADASIL). Neuropathol Appl Neurobiol 2015; 41: 557–570. 25303037 10.1111/nan.12188 PMC5098250

[45] Miners S Moulding H de Silva R et al. Reduced vascular endothelial growth factor and capillary density in the occipital cortex in dementia with Lewy bodies. Brain Pathol 2014; 24: 334–343. 24521289 10.1111/bpa.12130 PMC8029164

[46] Verbeek MM Van Nostrand WE Otte-Holler I et al. Amyloid-β-induced degeneration of human brain pericytes is dependent on the apolipoprotein E genotype. Ann N Y Acad Sci 2000; 903: 187–199. 10818507 10.1111/j.1749-6632.2000.tb06368.x

[47] Love S Miners JS Cerebral hypoperfusion and the energy deficit in Alzheimer's disease. Brain Pathol 2016; 26: 607–617. 27327656 10.1111/bpa.12401 PMC8028913

[48] Masliah E Mallory M Alford M et al. PDGF is associated with neuronal and glial alterations of Alzheimer's disease. Neurobiol Aging 1995; 16: 549–556. 8544904 10.1016/0197-4580(95)00050-o

[49] Yang SP Bae DG Kang HJ et al. Co-accumulation of vascular endothelial growth factor with beta-amyloid in the brain of patients with Alzheimer's disease. Neurobiol Aging 2004; 25: 283–290. 15123332 10.1016/S0197-4580(03)00111-8

[50] Hagedorn M Balke M Schmidt A et al. VEGF coordinates interaction of pericytes and endothelial cells during vasculogenesis and experimental angiogenesis. Dev Dynam 2004; 230: 23–33. 10.1002/dvdy.20020 15108306

[51] Greenberg JI Shields DJ Barillas SG et al. A role for VEGF as a negative regulator of pericyte function and vessel maturation. Nature 2008; 456: 809–813. 18997771 10.1038/nature07424 PMC2605188

[52] Bergers G Song S The role of pericytes in blood-vessel formation and maintenance. Neuro-Oncology 2005; 7: 452–464. 16212810 10.1215/S1152851705000232 PMC1871727

[53] Bai Y Zhu XJ Chao J et al. Pericytes contribute to the disruption of the cerebral endothelial barrier via increasing VEGF expression: Implications for stroke. Plos One 2015; 10: e0124362. 25884837 10.1371/journal.pone.0124362 PMC4401453

[54] Patel NS Mathura VS Bachmeier C et al. Alzheimer's beta-amyloid peptide blocks vascular endothelial growth factor mediated signaling via direct interaction with VEGFR-2. J Neurochem 2010; 112: 66–76. 19818105 10.1111/j.1471-4159.2009.06426.x

[55] Charidimou A Pantoni L Love S The concept of sporadic cerebral small vessel disease: A road map on key definitions and current concepts. Int J Stroke 2016; 11: 6–18. 26763016 10.1177/1747493015607485

[56] Ashby EL Love S Kehoe PG Assessment of activation of the plasma Kallikrein-Kinin system in frontal and temporal cortex in Alzheimer's disease and vascular dementia. Neurobiol Aging 2012; 33: 1345–1355. 21074291 10.1016/j.neurobiolaging.2010.09.024

[57] Bartus RT Elliott P Hayward N et al. Permeability of the blood brain barrier by the bradykinin agonist, RMP-7: Evidence for a sensitive, auto-regulated, receptor-mediated system. Immunopharmacology 1996; 33: 270–278. 8856161 10.1016/0162-3109(96)00070-7

[58] Bartus RT Elliott PJ Dean RL et al. Controlled modulation of BBB permeability using the bradykinin agonist, RMP-7. Exp Neurol 1996; 142: 14–28. 8912895 10.1006/exnr.1996.0175

[59] Raymond JJ Robertson DM Dinsdale HB Pharmacological modification of bradykinin induced breakdown of the blood-brain barrier. Can J Neurol Sci 1986; 13: 214–220. 3742336 10.1017/s0317167100036301

