# Supplementary material for: Differing associations between Aβ accumulation, hypoperfusion, blood–brain barrier dysfunction and loss of PDGFRB pericyte marker in the precuneus and parietal white matter in Alzheimer's disease
Source: J Cereb Blood Flow Metab. 2017 Feb 2;38(1):103–15. doi: 10.1177/0271678X17690761 (PMC5757436; doi:10.1177/0271678X17690761)
Supplement: Supplementary material [file JCB690761_supplementary_material.pdf]

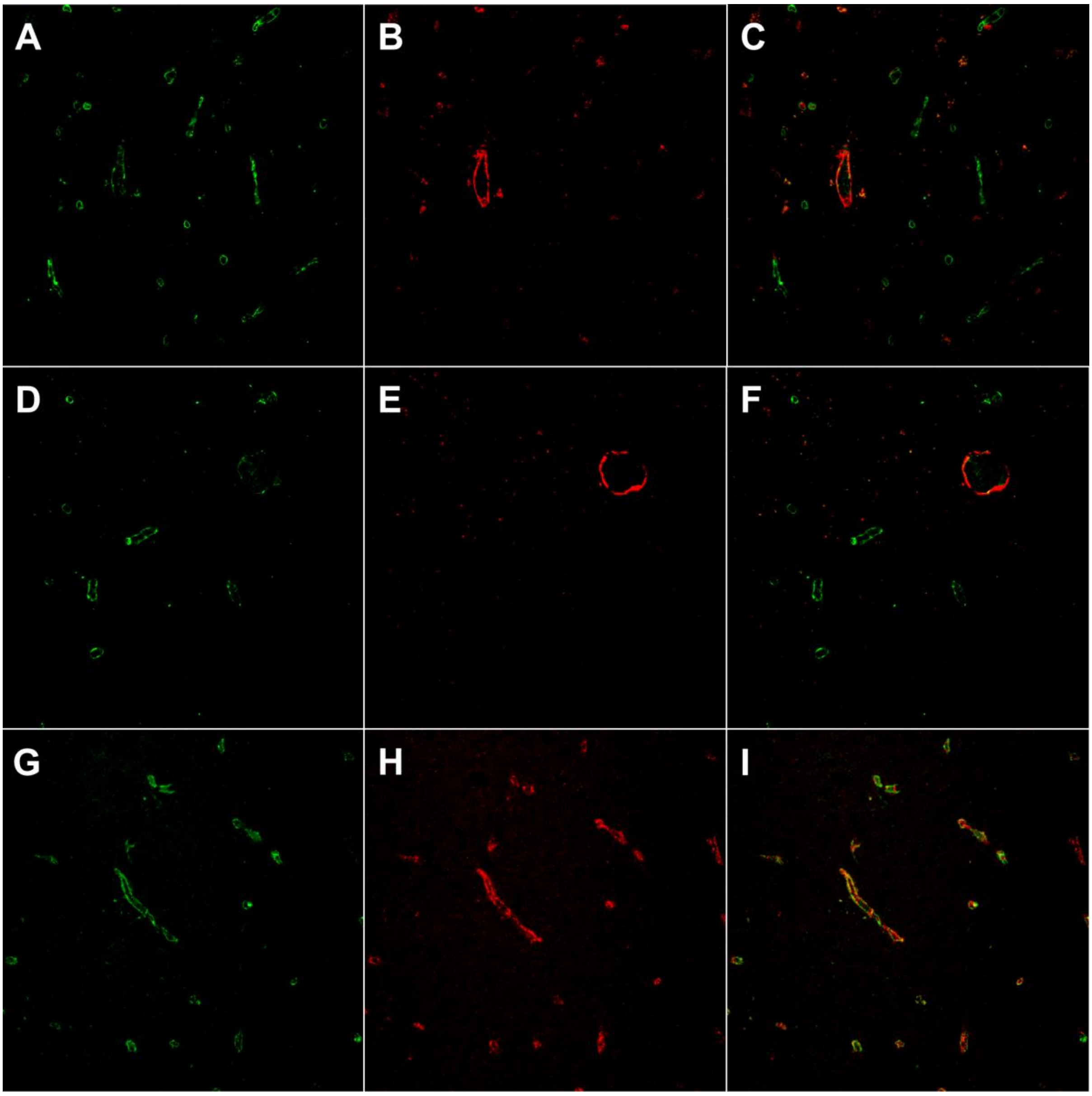

Supplementary Figure 1. Immunolocalization of PDGFRB to capillaries. Sections of parietal cortex (**A-C**) and white matter (**D-F**) were immunolabelled with antibodies to PDGFRB (green signal) and smooth muscle actin (red signal). PDGFRB was almost entirely restricted to capillaries. There was minimal overlap of PDGFRB signal with that of smooth muscle actin in adjacent arterioles (see combined labeling in C and F). Sections of cortex (**G-I**) were also immunolabeled for PDGFRB (green signal) and vWF (red signal). Both PDGFRB and vWF (expressed by endothelial cells) localize to capillaries, with limited overlap of the two antigens (see I). PDGFRB in all of these sections was immunolabelled with the same goat anti-PDGFRB detection antibody that was used for ELISA detection of PDGFRB in the brain homogenates (duoset, Cat no DYC385, R&D systems, Oxford, UK). After rehydration the paraffin sections were microwaved for 10 min in Tris-EDTA buffer (10 mM Tris, 1mM EDTA, pH 9.0), rinsed, and incubated at 4°C overnight with the primary antibodies (biotinylated goat polyclonal anti-human PDGFRB, at 0.9 µg/ml together with either rabbit polyclonal anti-human smooth muscle actin, Abcam, Cat no ab5694, Cambridge, UK, at 0.4 µg/ml, or rabbit polyclonal anti-vWF, Dako, Glostrup, Denmark, at 155 µg/ml) in PBS containing 0.1%

triton X-100 and 10% donkey serum. After thorough rinsing of the sections, bound antibody was detected by incubation for 1 h at room temperature with streptavidin Alexa-Fluor 488 (Molecular Probes, Invitrogen) and Alexa Fluor 568 donkey anti-rabbit (Molecular Probes, Invitrogen) diluted 1:200 in PBS. The sections were mounted in Vectashield Mounting medium containing DAPI (Vector Labs, H-1200, Peterborough, UK).

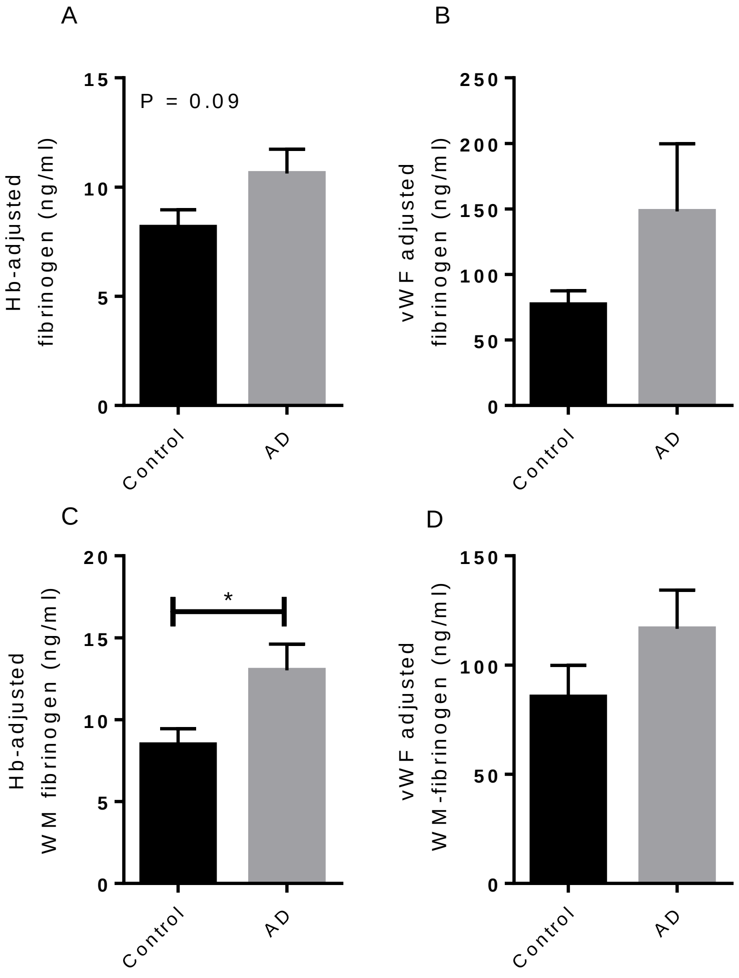

Supplementary Figure 2. Fibrinogen levels are increased in the precuneus and underlying white matter in Alzheimer's disease after adjustment for hemoglobin level and vessel density. (**A-B**) Bar charts showing a trend towards increased fibrinogen in the precuneus after adjustment for hemoglobin level (adjusted for total hemoglobin content within brain tissue) and vessel density (adjusted for von Willebrand factor (vVWF) content). (**C-D**) Fibrinogen level was significantly increased in the underlying white matter in AD after adjustment for total hemoglobin level ($p < 0.05$) and for vessel density although not significantly. The bars indicate the mean and SEM. *$P < 0.05$

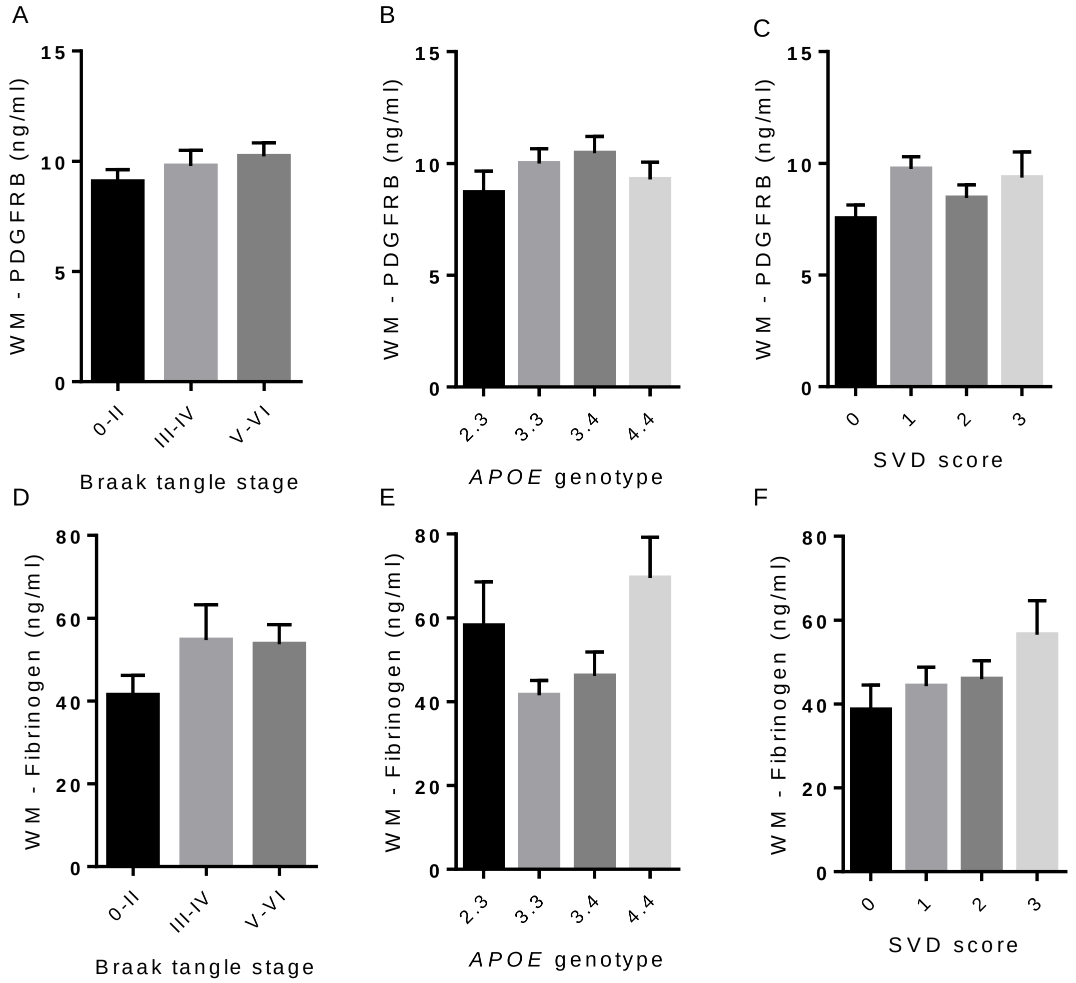

Supplementary Figure 3. Platelet-derived growth factor receptor-β (PDGFRB) loss and blood-brain barrier (BBB) breakdown in relation to disease severity (i.e. Braak tangle stage), *APOE* genotype, and cerebral amyloid angiopathy (CAA) in the precuneus in AD. Bar charts showing non-significant trends towards increased white-matter (WM) PDGFRβ level in relation to (**A**) disease severity when cases were grouped according to Braak tangle stage and (**B**) *APOE* genotype and (**C**) SVD score. Bar charts showing non-significant trends towards increased WM-fibrinogen level in relation to (**D**) disease severity when cases were grouped according and (**E**) *APOE* genotype and (**F**) SVD score. The bars indicate the mean and SEM. SVD score based on a 4-point semi-quantitative scale as previously described [23], according to the extent of thickening of the arteriolar walls and associated narrowing of the vessel lumina: 0 = normal vessel wall thickness, 1 = slightly increased thickness, 2 =

moderately increased thickness, and 3 = markedly increased thickness such that for many arterioles the diameter of the lumen was <50% of the outer diameter of the blood vessel.

Supplementary Table 1

		**AD** (n = 49)	**Control** (n = 37)
**Age (y ± SD)**		77.5 ± 8.2	79.8 ± 8.9
**Gender (F:M)**		26:23	11:26
**PM delay (h ± SD)**		31.4 ± 19.3	32.7 ± 16.3
**Braak tangle stage**	0	0	4
	I	0	6
	II	0	19
	III	0	6
	IV	2	0
	V	20	0
	VI	27	0
**APOE genotype**	2.3	2	11
	3.3	12	19
	3.4	22	7
	4.4	12	0
**SVD score**[#]	0	6	7
	1	21	18
	2	17	8
	3	5	0
**CAA score***	0	15	25
	1	9	3
	2	10	4
	3	15	1

Supplementary Table 2

Diagnosis	MRC Identifier	Age-at-death (y)	Post-mortem delay (h)	Gender	Braak tangle stage	APOE genotype	SVD score	CAA score
Control	BBN_8651	95	46	F	2	2.3		
Control	BBN_8671	78	24	F	2	3.3	2	1
Control	BBN_8700	64	12	M	2	2.3	0	0
Control	BBN_8702	58	20	M	0	2.3	0	0
Control	BBN_8706	72	42	M	1	3.3	1	0
Control	BBN_8708	90	45	M	2	2.3	0	0
Control	BBN_8717	77	55	M	1	3.3	1	0
Control	BBN_8722	78	12	M	2	3.3	1	0
Control	BBN_8723	80	67	M	3	3.4	2	2
Control	BBN_8725	73	36	M	2	3.4	0	0
Control	BBN_8739	93	18	F	2	3.3	1	0
Control	BBN_8751	82	30	M	2	3.3	2	0
Control	BBN_8759	75	48	M	2	3.3	1	0
Control	BBN_8776	73	33	M	1	2.3	1	0
Control	BBN_8779	69	66	M	2	3.3	1	0
Control	BBN_8835	73	59	F	1	3.3	1	0
Control	BBN_8883	90	40	M	3	3.3	1	0
Control	BBN_8898	83	24	F	2	3.4	0	0
Control	BBN_8923	82	3	M	2	3.3	1	0
Control	BBN_8949	79	24	M		3.4		
Control	BBN_8957	76	12	F		3.4		
Control	BBN_8980	72	24	F	0	3.3	2	0
Control	BBN_8983	78	48	M	1	3.3	1	0
Control	BBN_9028	76	23	M	2	3.3	1	0
Control	BBN_9092	75	6	M	3	2.3		
Control	BBN_9292	73	35	M	3	3.3	1	0
Control	BBN_9299	90	5.5	M	2	2.3	1	0
Control	BBN_9311	93	37.75	M	3	2.3	2	1
Control	BBN_9292	80	45.75	M	0	3.3	2	2
Control	BBN_9340	94	21	F	2	2.3	2	2

Control	BBN_9346	92	34.25	M	2	3.4	2	0
Control	BBN_4206	87	24	M	2	3.3	1	1
Control	BBN_9354	85	30.5	M	2	3.3	0	0
Control	BBN_9359	77	42	M	1	3.3	1	2
Control	BBN_4229	87	47	F	3	2.3	1	0
Control	BBN_9365	86	32	F	2	3.4	1	3
Control	BBN_9389	68	38.75	F	0	2.3	0	0
AD	BBN_8834	78	9	F	5	3.4	2	0
AD	BBN_8848	77	43	F	4	3.4	0	0
AD	BBN_8910	71	30	M	6	3.3	2	2
AD	BBN_8912	82	24	F	6	3.4	1	3
AD	BBN_8921	75	40	F	6	3.3	1	3
AD	BBN_8997	74	12	F	6	3.4	1	3
AD	BBN_9005	89	4	F	6	3.4	1	3
AD	BBN_9026	79	28	M	6	3.4	1	1
AD	BBN_9030	65	27	M	6	3.4	1	2
AD	BBN_9031	85	66	M	6	3.4	1	3
AD	BBN_9044	86	48	M	6	4.4	2	0
AD	BBN_9052	57	24	F	5	3.4	1	0
AD	BBN_9076	84	20	F	5	3.4	3	0
AD	BBN_9106	93	20	M	6	3.3	1	0
AD	BBN_9112	74	52.5	F	5	4.4	1	3
AD	BBN_9122	83	5	F	5	3.4	2	0
AD	BBN_9123	74	35	F	5	3.3	2	3
AD	BBN_9136	77	26	F	6	3.4	1	3
AD	BBN_9155	79	27	M	6	3.4	2	1
AD	BBN_9162	63	43	M	6	3.3	0	0
AD	BBN_9163	69	71	F	6	3.4	1	3
AD	BBN_9164	92	24	F	5	3.3	0	3
AD	BBN_9173	86	31	F	5	3.4	2	2
AD	BBN_9179	64	9	M	6	3.4	1	1
AD	BBN_9181	80	48	F	5	4.4	1	2
AD	BBN_9182	74	24	M	5	3.4	2	1
AD	BBN_9186	75	21	F	6	3.4	1	1

AD	BBN_9189	78	21	F	6	4.4	2	3
AD	BBN_9194	89	39	F	5	4.4	2	3
AD	BBN_9197	77	14	F	6	3.4	2	0
AD	BBN_9205	85	85	F	6	3.4	2	3
AD	BBN_9261	83	48	M	5	3.3	2	2
AD	BBN_9262	81	4	M	6	4.4	2	3
AD	BBN_9263	74	48	M	5	2.3	3	1
AD	BBN_9266	80	72	M	5	3.3	1	2
AD	BBN_9274	78	49	M	6	4.4	0	2
AD	BBN_9275	87	36	M	6	4.4	1	0
AD	BBN_9280	76	11	M	5	4.4	3	0
AD	BBN_9295	85	49.5	M	7	3.3	3	0
AD	BBN_9303	69	12	M	5	3.4	1	0
AD	BBN_9315	67	24.25	F	6	4.4	2	1
AD	BBN_9323	84	20.5	F	6	2.3	3	2
AD	BBN_9342	65	11.5	F	6	4.4	1	2
AD	BBN_4202	64	66.5	M	5	3.3	0	0
AD	BBN_4204	65	38.5	M	5	3.3	2	0
AD	BBN_4215	80	26	F	4	3.4	0	2
AD	BBN_9361	83	11	M	5	3.3	1	1
AD	BBN_9367	77	19	M	6	4.4	1	3
AD	BBN_9378	84	22	F	5		2	1

SVD – small vessel disease; CAA - Cerebral amyloid angiopathy

Supplementary Table 3. Correlations between platelet-derived growth factor-ββ (PDGF-BB) level and soluble and insoluble Aβ40 and Aβ42 levels within the precuneus and underlying white matter in SDS-extracted (soluble) and guandine-HCl-extracted (insoluble) extracts of brain tissue.

Precuneus:	**soluble Aβ40**	**soluble Aβ42**	**insoluble Aβ40**	**insoluble Aβ42**
PDGFBB (soluble)	†NS	NS	NS	NS
PDGFBB (insoluble)	NS	0.254*	0.430**	0.400**
**White matter:**	**soluble Aβ40**	**soluble Aβ42**	**insoluble Aβ40**	**insoluble Aβ42**
PDGFBB (soluble)	NS	0.304**	NS	0.264*
PDGFBB (insoluble)	NS	NS	-0.298*	-0.212*

†Not significant, * P < 0.05, ** P < 0.01
